# Use of Isoniazid Monotherapy in Comparison to Rifamycin-Based Regimen for the Treatment of Patients With Latent Tuberculosis: A Systematic Review

**DOI:** 10.7759/cureus.25083

**Published:** 2022-05-17

**Authors:** Noor ul ain Shahid, Noreen Naguit, Rakesh Jakkoju, Sadia Laeeq, Tiba Reghefaoui, Hafsa Zahoor, Ji Hyun Yook, Muneeba Rizwan, Lubna Mohammed

**Affiliations:** 1 Department of Research, California Institute of Behavioral Neurosciences & Psychology, Fairfield, USA

**Keywords:** latent tuberculosis, isoniazid, rifamycin, rifapentine, rifampin, latent tuberculosis treatment, latent tuberculosis infection

## Abstract

Tuberculosis (TB) is a common infectious disease that is present all around the world. This insidious disease needs drastic measures for its eradication. One of the actions contributing to it is the timely diagnosis and offering suitable treatment options for latent tuberculosis patients. In this review, we will discuss and compare the variety of options available for this purpose. We searched PubMed/Medline, Cochrane library, Google Scholar, and Science Direct to find articles regarding the effectiveness, safety, and completion of any of the five regimens available for latent tuberculosis infection. These options are the most classic and standard nine months of isoniazid given daily, which is now more commonly given as six months course, three months of daily isoniazid and rifampin, three months of weekly isoniazid and rifapentine, and four months of daily rifampin. We looked into free full-text studies published from 2011 to 2021 available in English language and human studies. After applying inclusion/exclusion criteria and removing duplicates and screening, 34 articles were shortlisted for quality assessment check, after which we finalized nine studies. Cochrane risk-of-bias assessment tool was used for quality check of randomized control trials, New-Castle Ottawa tool for observational studies, and assessment of multiple systematic reviews (AMSTAR) tool for systematic reviews. Efficacy was checked by tracking down the new cases of TB in the sample population that took the treatment for latent tuberculosis infection. New rifamycin-based regimens were almost equal in effectiveness to isoniazid regimens. The side effect profile is different for both regimens, but short-duration courses tend to have a higher chance of completion.

## Introduction and background

Tuberculosis (TB) is one of the deadliest infectious diseases known worldwide [1]. Approximately 1.7 to 2 billion people are estimated to be infected with mycobacterium tuberculosis bacteria (Mtb) [2,3]. In other words, one-third to one-fourth of the world's population gets infected with these bacteria [4]. Not all individuals infected with the TB bacteria become sick or develop the active disease [4]. But these individuals are a pool of potential new cases in coming years. Statistics state that 5-10% of them will develop the active disease due to the inability of their immune system to fight or constrain bacteria, as a result of which bacteria start multiplying in the body, producing symptoms like fever and night sweats. In those infected, 10% can fight off and get rid of bacteria completely, whereas 90% can limit the spread of bacteria incarcerated in a confined space by protective mechanisms of the body. This process stops them from growing, and it becomes inactive [5]. However, bacteria keep living in the body without making you sick. This is called latent tuberculosis infection (LTBI) [4]. Immune responses are continuously fighting against it. Therefore people with LTBI are symptomless and cannot spread the infection to others as opposed to people with the active TB disease who are contagious until up to four weeks of treatment [4].

World Health Organizations' (WHO) "END TB" strategy aims to reduce TB-related mortality by 95% and TB-related new cases by 90% by 2035 [1]. To achieve that target, timely identification of the patients with latent tuberculosis and giving them suitable treatment options to ensure compliance is crucial [1]. The high-risk population should especially undergo monitoring regularly. That includes immunocompromised people, individuals with comorbidities like AIDS and diabetes, people going through dialysis, or organ transplant recipients [6,7]. Since there are no positive findings in the chest x-ray of patients with LTBI, the two diagnostic tests frequently used to shortlist these patients are the tuberculin skin test (TST) and the interferon-gamma release assay (IGRA) [5]. These tests detect the immune responses of the body to Mtb antigens [4]. The interferon-gamma test includes the following three T-SPOT TB, quantification TB Gold, and QuantiFERON TB Gold in the tube.

While the active disease treatment involves a combination of four drugs, isoniazid (INH), rifampin (RFMP), pyrazinamide (PZA), and ethambutol (EMB), due to a more significant bacterial load which are continuously multiplying, the treatment of latent infection comprises of one or a combination of two drugs [8]. The earliest and the most commonly used treatment for LTBI is isoniazid for nine months [9]. If full treatment is completed, it prevents latent TB from becoming active by 85-90% [9,10]. While the long duration of therapy increases its protective efficacy, the treatment completion rate becomes low [10,11]. It has been estimated that nine months of isoniazid have a 50% or less completion rate [9]. Additionally, the longer treatment duration with INH is associated with the adverse effects of hepatotoxicity and neuropathy [10]. Isoniazid for six months is also an accepted regimen in adults and children due to better adherence by the patients [11,12].

In 2010, multiple randomized control trial (RCT) findings led the Centres for Disease Control and Prevention (CDC) to approve another treatment for LTBI but by directly observed therapy (DOT) only [2], which was isoniazid and rifapentine once weekly for three to four months (3HP) [4]. Rifapentine is as effective as rifampin but with a much longer half-life, hence once weekly dosing [13]. But this regimen did not have enough evidence of safety and efficacy for its use in AIDS patients and children [2]. The fourth treatment option recommended by WHO is three to four months of isoniazid and rifampin taken daily (INH/RFMP), and the last one is four months of daily rifampin alone (RFMP) [4]. Generally, four months of rifampicin is considered a second choice compared to other regimens. Still, when chosen appropriately in a suitable population, it has significant completion rates and has a low cost to health care settings [10]. A previous trial named "the prevent TB clinical trial" and a few other clinical trials have shown higher treatment completion with 3HP DOT therapy than self-administered isoniazid therapy of nine months [3,14].

Daily isoniazid is recommended for nine months to be taken in a dose of 5 mg/kg body weight, rounded to the nearest 50 mg or 100 mg, with a maximum of 300 mg according to the American Thoracic Society (ATS) and Infectious Disease Society of America (IDSA) and CDC clinical practice guidelines [9,15]. For three months of isoniazid and rifapentine (RPT), INH at a dose of 15 mg/kg with a maximum dose of 900 mg and RPT at 300-900 mg based on weight, given in the form of once-weekly 12 dosing schedules under directly observed therapy [9] and 120 doses of daily rifampin at 10 mg/kg up to 600 mg for four months of rifampin regimen (4R) [9,15].

Given the multiple treatment options for latent TB, health care providers and infectious disease control doctors have a choice to select the best possible and suitable regimen for the patient, based on the individual's background, exposure, commitment to complete the treatment, cost-effectiveness, chosen regimen's efficacy, associated adverse effects, and what other reactions these drugs can produce on that particular patient's health. This systematic review compares the effectiveness-drug-associated harmful effects and completion rates of isoniazid-based regimens with rifamycin-based regimens. We aimed to deliver a comprehensive understanding of available options more simply and efficiently for the convenience of doctors. Evidence-based informed choices can aid in the quicker eradication of TB.

Multiple studies on latent tuberculosis treatment comparisons are primarily performed on HIV-positive patients, immunocompromised patients, or patients who are more prone to developing TB due to the intake of immunosuppressant drugs. However, latent tuberculosis treatment in non-HIV otherwise healthy people is a lesser-explored part of this field and needs work on its latest updates. To our knowledge, a few systematic reviews were also done, mainly focusing on INH - rifapentine option [4,5]. In 2013, the Cochrane systematic review concluded that the rifampicin-based treatment option might have lesser side effects and better completion rates [5].

Methods

This systematic review and its reporting of results use the Preferred Reporting Items for Systematic Reviews and Meta-Analysis (PRISMA) guidelines and principles [16].

Search Strategy

We developed an expanded search strategy to select all the studies that provided information on different types of latent tuberculosis treatment, their comparison with one another, their individual and comparative efficacy, as well as adverse effects related to any of the drugs used for the treatment of tuberculosis. In addition, we also selected the data and the studies that reported completion rates and compliance, reasons for the failure of treatment in any of the five available regimens, which are (1) isoniazid alone for nine months, (2) isoniazid alone for six months, (3) isoniazid and rifapentine once weekly for three months, (4) isoniazid and rifampin daily for three months, and (5) rifampin daily for four months. While conducting the data search, we used controlled vocabulary and medical subject headings (MeSH) keywords. Controlled vocabulary was adjusted while searching through various databases.

Databases and Keywords Used

We searched PubMed/Medline, Cochrane library, Google Scholar, and Science Direct, including gray literature elements from 2011-to 2021. The keywords used were latent tuberculosis, latent tuberculosis treatment, rifampin, rifapentine, rifamycin, isoniazid, and latent tuberculosis treatment comparison between isoniazid and rifampin.

MeSH Strategy for PubMed

MeSH keywords used were latent tuberculosis OR latent tuberculosis treatment OR ("latent tuberculosis"{MeSH}) OR (“latent tuberculosis/drug therapy"{MeSH} OR "latent tuberculosis/metabolism"{MeSH} OR "latent tuberculosis/mortality"{MeSH} OR "latent tuberculosis/organization and administration"{MeSH} OR "latent tuberculosis/physiopathology"{MeSH} OR "latent tuberculosis/prevention and control"{MeSH} OR "latent tuberculosis/statistics and numerical data"{MeSH} OR "latent tuberculosis/therapy"{MeSH} OR "latent tuberculosis/urine"{MeSH}) AND rifamycin OR rifapentine OR rifampin OR ("rifamycins"{Majr}) AND (“rifamycins/administration and dosage"{Majr} OR "rifamycins/adverse effects"{Majr} OR "rifamycins/metabolism"{Majr} OR "rifamycins/organization and administration"{Majr} OR "rifamycins/pharmacokinetics"{Majr} OR "rifamycins/statistics and numerical data"{Majr} OR "rifamycins/therapeutic use"{Majr} OR "rifamycins/therapy"{Majr} OR "rifamycins/toxicity"{Majr} OR "rifamycins/urine"{Majr}) AND isoniazid OR ("isoniazid"{Majr}) OR (“isoniazid/administration and dosage"{Majr} OR "isoniazid/adverse effects"{Majr} OR "isoniazid/metabolism"{Majr} OR "isoniazid/organization and administration"{Majr} OR "isoniazid/pharmacokinetics"{Majr} OR "isoniazid/statistics and numerical data"{Majr} OR "isoniazid/therapeutic use"{Majr} OR "isoniazid/therapy"{Majr} OR "isoniazid/toxicity"{Majr} OR "isoniazid/urine"{Majr}).

Inclusion/Exclusion Criteria 

Free full-text articles from 2011 to 2021 in English language, human studies, all types of studies (case-control, RCT, observational cohort, reviews, systematic reviews, and meta-analysis), and gray literature elements from 2011 to 2021 were included in this study. Age less than two years, people diagnosed with comorbidities like HIV, rheumatoid arthritis, organ transplant recipients, and patients on dialysis receiving Tb prophylactic treatment were excluded.

Study Selection

We looked into gray literature as well and included guidelines and updates regarding LTBI treatment and recommendations. The study population consists of age ranges (>12 years, 2-12 years) diagnosed with LTBI by either TST or IGRA. Initially, people diagnosed with comorbidities like HIV, rheumatoid arthritis, organ transplant recipients, and patients on dialysis receiving prophylaxis for TB were part of our inclusion criteria but were later excluded in the "screening section." Outcomes reported were rate of completion of treatment, drug-related adverse effects, effectivity, and causes stating reasons for discontinuation of treatment of any of the available treatment regimens for latent tuberculosis. The last date of the database search was September 1, 2021.

Analysis of Study Quality

We checked included RCTs through the Cochrane risk of bias tool and summarized the results of each domain assessed as a judgment (high, low, or unclear). The assessment results of four randomized control trials are shown below in Table 1.

**Table 1:** A tabulated form of Cochrane risk of bias tool results for included randomized control trials

Domain	Menzies et al. [11]	Sterling et al. [3]	Sun et al. [1]	Surey et al. [6]
Selection bias: random sequence generation	Low risk	Low risk	Low risk	Low risk
Selection bias: allocation concealment	Low risk	Low risk	Low risk	Low risk
Reporting bias: selective reporting	Low risk	Low risk	Unclear	Low risk
Detection bias: blinding outcome assessment	Low risk	Unclear	No information	Low risk
Performance bias: blinding participants and personnel	Unclear	Unclear	No information	No information
Attrition bias: incomplete outcome data	Low risk	Low risk	Low risk	Low risk
Other bias: other sources of bias	Low risk	Unclear	Low risk	Low risk

Our final studies included observational studies as well. They were gone through quality appraisal by the New-Castle Ottawa risk-of-bias tool where each study was checked for three domains of selection, comparability, and outcome of results, and then the final score was given. A sum score of nine to seven was considered low risk, six to four was considered a moderate risk, and three to zero was considered a high risk of bias. The results of our final included studies are given below in Table 2. 

**Table 2:** New-Castle Ottawa risk-of-bias tool results for included observational studies A sum score of 9 -7 is considered low risk, 6-4 is considered moderate risk, and 3-0 is high risk.

Study	Selection	Comparability	Outcome/exposure	Sum score	Risk
Maracaig et al. [9]	3	1	3	7	Low
McClintock et al. [10]	3	1	3	7	Low
Fröberg et al. [13]	3	0	3	6	Moderate

Systematic reviews were checked for quality by assessment of multiple systematic reviews (AMSTAR) guidelines where different components of the study were subject to a questionnaire and the results of the final studies are summarized below in Table 3*.*

**Table 3:** AMSTAR checklist results for included systematic reviews PICO: population, intervention, comparability, outcome; ROB: risk of bias; AMSTAR: assessment of multiple systematic reviews

Question	Sharma et al. [5]			Peace et al. [4]		
	Yes	Partial yes	No	Yes	Partial yes	No
1. Did the research questions and inclusion criteria for the review include the components of PICO?	Yes			Yes		
2. Did the review report contain a clear declaration that the review methods were established before the review was done, and did the report justify any significant deviations from the protocol?		Partial yes			Partial yes	
3. Did the review authors explain their selection of the study designs for inclusion in the review?	Yes			Yes		
4. Did the review authors use a comprehensive literature search strategy?	Yes			Yes		
5. Did the review authors perform study selection in duplicate?	Yes					No
6. Did the review authors perform data extraction in duplicate?	Yes			Yes		
7. Did the review authors provide a list of excluded studies and justify the exclusion?	Yes			Yes		
8. Did the review authors describe the included studies in adequate detail?	Yes			Yes		
9. Did the review authors use a satisfactory technique for assessing the risk of bias in individual studies that were included in the review?	Yes			Yes		
10. Did the review authors report on the sources of funding for the studies included in the review?			No			No
11. If meta-analysis was performed, did the review authors use appropriate methods for statistical combination of results?	Yes			Yes		
12. If meta-analysis was performed, did the review authors assess the potential impact of ROB in individual studies on the meta-analysis results or other evidence synthesis?		Partial yes			Partial yes	
13. Did the review authors account for ROB in individual studies when interpreting/discussing the review results?	Yes			Yes		
14. Did the review authors provide a satisfactory explanation for, and discussion of, any heterogeneity observed in the results of the review?	Yes			Yes		
15. If they performed quantitative synthesis, did the review authors carry out sufficient investigation of publication bias and debated its most likely impact on the results of the review?	Yes					No
16. Did the review authors report any potential sources of conflict of interest, including any funding they received for conducting the review?			No	Yes		

Results

**Figure 1:**
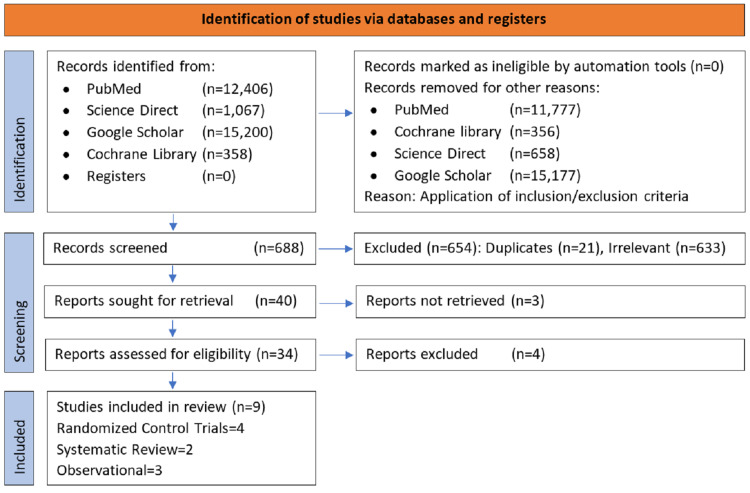
PRISMA flow diagram showing the process of studies selection PRISMA: Preferred Reporting Items for Systematic Reviews and Meta-Analyses

We retrieved 29,031 studies by our searches performed on the databases mentioned above published from 2011 to 2021. After applying inclusion/exclusion criteria to the studies of each database, we finalized 629 articles from PubMed/Medline. Two hundred and fifty-eight articles from Cochrane Library, 14,800 from Google Scholar, and 692 from Science Direct. We reviewed them for eligibility criteria again, shortlisted a total of 688 articles for screening, and removed 21 duplicates at this stage. The remaining were accessed by going through titles and abstracts. Most relevant articles were selected that precisely reported on the effectiveness, toxicity, and compliance of any of the four treatment regimens for LTBI. The final 34, most pertinent articles consisted of eleven observational studies, ten randomized control trials, seven systematic reviews/meta-analyses, and six others (gray literature, prescription data extraction from registers, editorials). Quality assessment was performed on the above studies using Cochrane risk of a bias assessment tool for randomized control trials, New-Castle Ottawa risk-of-bias assessment tool for observational studies, and "AMSTAR" guidelines for systematic review and meta-analysis. Quality appraisal was checked by two authors separately, and disagreements were resolved by consensus. The flow diagram below depicts this procedure of data selection in Figure 1.

Table 4 below shows the important features like the year of publication, sample size, type of study, population characteristics, outcome, interventions used, and conclusion of studies by authors in summarized form.

**Table 4:** A tabulated form of final studies included Grade 3-5 adverse events - serious adverse events that may result in death, is life threatening, requires patient to stay in hospital for longer duration or increase the stay if already hospitalized, results in persistent or significant disability/incapacity or birth defects [11]. RCT: randomized control trials; TST: tuberculin skin test; IGRA: interferon-gamma release assay; TB: tuberculosis; 9INH group: nine months of daily isoniazid group; 4RFMP group: four months of daily rifampin group; 3HP group: three months of weekly isoniazid and rifapentine; DOT: directly observed therapy; 3INH/RFMP group: three months of daily isoniazid and rifampin group; LTBI: latent tuberculosis infection; SAD: self-administered therapy; NYC: New York City; INH/RPT-3: weekly isoniazid and rifapentine for three months; INH-9: daily isoniazid for nine months; INH-6: daily isoniazid for six months; INH/RFMP 3-4: daily isoniazid and rifampin for three to four months; RFMP-4: daily rifampin for four months; LFTs: liver function tests

Author and year of publication	Sample size	Study type	Study population/type	Aim of study/outcome	Interventions	Conclusion
Sterling et al., 2011 [3]	3986 patients in 3HP group; 3745 patients in isoniazid-only group.	Prospective open-label RCT	June 2001-February 2008; 12 or greater years of age or 2-11 years of age who had close contact with culture-positive TB pt. and has a positive TST. HIV patients with positive TST. Just had a close contact positive TST with chest x-ray fibrotic changes.	Primary outcome: culture-confirmed TB in 18 or greater years of age, culture-confirmed or clinical TB in 18 or fewer years of age. Secondary outcome: completion of study therapy, permanent discontinuation of treatment, permanent discontinuation because of adverse drug reaction grade 3 or 4.	Three months of once weekly rifapentine + isoniazid given under dot (3HP). Nine months of self-administered daily isoniazid (9INH).	3HP and 9INH had almost equal efficacy, but 3HP had more completion rates. The incidence of new cases of TB in the 9INH group was almost twice that of the 3HP group. Both groups' toxicity profile was the same, with 3HP having low rates of liver damage cases. Adverse events leading to permanent discontinuation of therapy were comparatively low in the 3HP group. (4.9 vs 3.7 %)
Sharma et al., 2013 [5]	10 trials (n=10,717)	Systematic review	Multiple RCT involving HIV-negative people diagnosed to have LTBI	Primary outcome: rates of active TB. Secondary outcome: TB related death, All-cause death, Incidence of drug-resistant TB, adherence to treatment, adverse events	INH for 6-12M rifampicin or rifamycin containing drug combinations (any dose or duration)	Percentage of completing treatment course is most likely more with short duration rifamycin regimens whereas adverse events may be more minor. 3HP regimen has more completion rates and less tendency of liver damage. However, failure to complete therapy due to drug-related side effects is highly likely in rifamycin-based regimen than in isoniazid. rifampicin alone has not resulted in more cases of active TB in comparison to the lengthy INH regimen.
McClintock et al., 2017 [10]	87 patients in 3HP group; 82 patients in 4RFMP group; 224 patients in the 9INH group.	Retrospective cohort	18 years of age or above with positive TST/IGRA in combination with chest x-ray during 2009 and between July 1, 2013 and June 30, 2014.	Primary outcome: Treatment completion	Three months of once weekly isoniazid and rifapentine (3HP group) Four months of daily rifampicin (4RFMP group) Nine months of daily isoniazid (9INH group)	Patients on the 4RFMP regimen and 3HP regimen have more tendency to complete the treatment than patients on the 9INH regimen, whereas 4RFMP and 3HP have equal chances of the fulfillment of the therapy. 4RFMP regimen is comparatively less expensive for patients and health care units.
Peace et al., 2017 [4]	16 RCT (n=44,149) and 14 RCT (n=44,128) included in the analysis of efficacy and completion.	Systematic review	RCTs involving LTBI patients of any age and reporting on efficacy or completion rate of at least 1 of the regimens of interest. (INH/RPT-3, INH-9, INH-6, INH/RFMP-3-4, RFMP-4) patient's diagnosed with LTBI by + TST/IGRA.	Primary outcome: completion rate of different latent TB regimens and their efficacy.	INH/RPT-3 INH-9 INH-6 INH/RFMP 3-4 RFMP-4	In comparison to long-duration courses, a shorter duration of the treatment, for example, 3-4 months, predicts a greater percentage of full completion of the course. The benefits of rifamycin and isoniazid-based regimens are approximately the same.
Macaraig et al., 2018 [9]	55 patients in 9INH group; 269 patients in 4RFPM group; 125 patients in 3 HP group.	Retrospective cohort	All patients with positive TST registered in 4 NYC-based TB Clinics between January 1, 2015, and June 30, 2015.	Primary outcome: Treatment completion (12 doses within 16 weeks for 3HP group) (120 doses within six months for 4RFMP group) (270 doses within 12 months for 9INH group)	Three months of once weekly isoniazid and rifapentine (3HP with DOT) four months of daily rifampicin (4RFMP under SAD) nine months of daily isoniazid (9INH group)	73% of people in the 3HP and 4RFMP groups completed the treatment, whereas 49% of people in the 9INH group completed the treatment. When comparing 3HP with 4RFMP under programmed conditions, 3HP had a higher percentage of completion than 4RFMP (79% vs70%)
Menzies et al., 2018 [11]	3416 patients present in the isoniazid group; 3443 patients present in the rifampin group	Open-label parallel group RCT	18 years of age or older with positive TST or IGRA Test. If they fulfill the criteria for an increased chance of reactivation of active TB. If their provider recommended treatment with INH.	Primary outcome: to compare the rates of confirmed active TB in two groups among all eligible patients for 28 months after randomization. Secondary outcome: the combined incidence of adverse events of grades 3-5 occurring throughout therapy or within the max time allowed for the completion of the regimen.	Control-Oral isoniazid (5mg/kg) taken daily for nine months (9INH group) experimental-oral rifampin (10 mg/kg) taken daily for four months. (4RFMP group	4RFMP group had a higher completion rate and lower incidence of side effects, including hepatotoxic and grade 3 to 5 side effects; 9INH group, on the other hand, had lower completion rates. Hepatitis related to the drug was the most typical side effect of grade 3 or 4.
Sun et al., 2018 [1]	132 patients in 3HP group; 139 patients in 9H group.	Prospective open-label multicentre RCT	12 or greater years of age with positive TST within one month after unprotected exposure, from January 2014 to May 2016.	Primary outcome: treatment completion. Secondary outcome: incidence of severe adverse drug reactions in each group.	Three months of weekly isoniazid and rifapentine under DOT (3HP group) Nine months of daily self-administered isoniazid (9INH group)	Compared to the 9INH group, the completion rate of the 3HP group was more, but they have more likelihood to discontinue the treatment because of adverse reactions. The side effects experienced by this group were apart from liver damage and were mild self-limiting or got better after seeking medical help.
Fröberg et al., 2019 [13]	30 patients	Retrospective observational study	HIV-negative migrants with positive TST or positive Quantiferon in tube Gold from May 2015 to December 2017.	Primary outcome: adverse events, particularly LFTs and adherence to DOT.	Three months of weekly isoniazid and rifapentine under directly observed therapy	Almost 90% completion rate was achieved with 3HP. Drug-related side effects were not severe and self-limiting, like abdominal pain, nausea, dizziness, headache, and flu-like sickness. One case of kidney damage was reported, most likely due to a past severe adverse reaction to rifapentine, but that still calls for attention.
Surey et al., 2021 [6]	27 patients in 3HP group; 25 patients in 3INH/RFMP group	Open-label RCT	People 16-65 years of age who were diagnosed with having LTBI by positive TST or IGRA between March 2015 and January 2017 in the United Kingdom.	Primary outcome: treatment completion (taking more than 90% of prescribed doses of treatment)	Three months of once, weekly isoniazid and rifapentine (3HP group with SAD) Three months of daily rifampicin and isoniazid (3INH/RFMP group with SAD)	The frequency and severity of harmful reactions and completion rates were almost the same in both groups.

## Review

In this section, we will discuss the diagnosis of TB, risk factors that contribute towards the conversion of latent infection to active disease, five treatment options available and recommended by WHO for the treatment of LTBI, and their effectiveness, safety, and relative percentages of completion of each regimen in comparison to the others.

Diagnosis of latent TB

**Figure 2:**
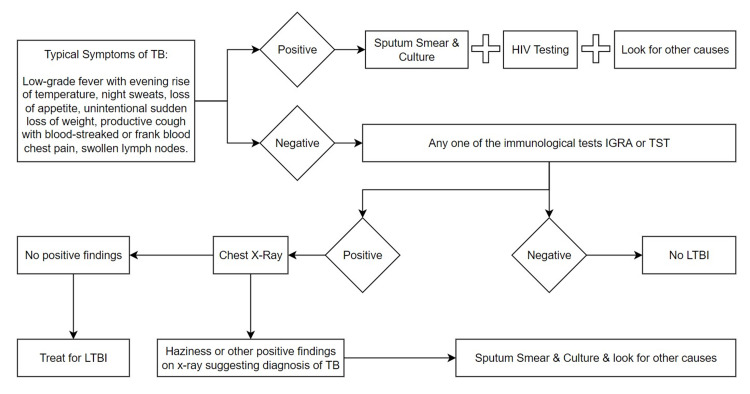
Flow diagram of investigations leading to the diagnosis of latent TB TB: tuberculosis; HIV: human immunodeficiency virus; IGRA: interferon-gamma release assay; TST: tuberculin skin test; LTBI: latent tuberculosis infection

Latent infection is an ongoing immune response to previously acquired bacteria but there is a small amount of bacterial load in latently infectious patients, therefore for the diagnosis of it, we depend on the immunity of the host rather than the bacteria itself [17,18]. The tuberculin skin test is a highly sensitive test for persons with normal immune responses and about 70% sensitive in persons with low immunity like those affected by HIV, but the specificity of the test is low. Interferon-gamma release essay test is reported to have high specificity in individuals with normal immunity. However, no difference from the tuberculin skin test was found in the results of immunocompromised patients. Because in the regions where TB is not common, most cases are due to TB reactivation in foreign-born individuals coming from areas where TB prevalence is high, predive values of both immunologic tests are of utmost importance and should be accessed completely [18]. In the absence of typical symptoms of TB like a persistent cough, fever, and night sweats, the patient is usually subjected to immunological tests and chest x-ray but in the presence of symptoms sputum smear and culture along with an x-ray are done. The flow diagram in Figure 2 is predicting the route and probable diagnosis of LTBI patients [17].

Risk factors for active TB disease

Suppressed immunity due to HIV or taking drugs that hinder a person's immune response like in organ transplant recipients or taking tumor necrosing factor alpha-blockers, diagnosed cases of silicosis and chronic renal failure (undergoing hemodialysis), close contact with a patient diagnosed with pulmonary tuberculosis are considered high-risk factors to develop an active TB disease from a latent infection [18]. Being a health care worker, diabetes, smoking, taking steroids, and having a fibronodular disease of the lung are other risk factors that should be considered [18].

Common adverse events related to TB drugs

To our consideration, an essential factor that makes a drug successful is its tolerability without causing any significant side effects to the patient. Various clinical trials and observational studies demonstrated the adverse impact of new vs. old regimens for LTBI treatment in multiple settings and with different age groups. Precautions should be taken when using rifamycin because irregular gaps between the dosing schedule could affect the efficacy and safety of the regimen. It is advisable to seek medical guidance at once if the patient develops yellow eyes, dizziness, fever, body aches, nausea, vomiting for greater than one day, weakness, abdominal pain, or loss of appetite [19]. Treatment can be retained while investigating the cause of symptoms. DOT workers should be adequately trained to check for signs from the adverse event checklist and involve the clinician when necessary [19].

Rifamycin is known to change the color of the secretions like urine and tears to red and cause hypersensitivity reactions such as headache, vertigo, pain in muscles and bones, fever, itching, and flu-like illness [19]. With the rifamycin use, especially rifapentine, intake of other drugs needs careful monitoring as it increases the metabolism of many medicines, particularly those that undergo their metabolism by cytochrome P450 isoenzyme 3A. Also, it is advisable to use a barrier method of contraception instead of hormonal if using any [19].

Treatment of LTBI

Just like any other clinical condition, tuberculosis has multiple treatment options available. The most classic 9H regimen has been used for over 50 years, but many new alternatives became available in the last decade with the latest advancements. Every regimen comes with its benefits and drawbacks that also change with the population group using it. What makes one regimen preferable over another is its effectiveness in a diverse population, including a wide range of races, ages, and health characteristics. However, prescribers need also to consider the potential tolerability of the drug, its cost-effectiveness, patient values, preferences, and feasibility that will ultimately affect their will to complete the treatment, based on the best quality evidence researchers can provide. The purpose of our research is to conduct a comparative study between different treatment regimens for latent tuberculosis that can offer quality evidence for clinicians and health professionals, especially public health officials and personnel, to make a better decision because all these efforts will help to achieve a TB-free world.

The recommended dose of isoniazid is 10 mg/kg/day in children and 5 mg/kg/day in adults with a maximum of 300 mg whereas rifampin in adults is also given as 10 mg/kg/day. Below in Table 5is the tabulated form of the WHO recommended treatment regimens for LTBI and recommended doses [18].

**Table 5:** Different treatment regimens for LTBI and recommended doses

Regimen	Dosage
Six months of isoniazid taken daily	Children: 10 mg/kg/day
	Adults: 5 mg/kg/day
	Maximum dose: 300 mg
Nine months of isoniazid taken daily	Children: 10 mg/kg/day
	Adults: 5 mg/kg/day
	Maximum dose: 300 mg
Four months of rifampin taken daily	Children: 10 mg/kg/day
	Adults: 10 mg/kg/day
	Maximum dose: 600mg
Three months of rifampin and isoniazid taken daily	Rifampin - children: 10 mg/kg/day
	Adults: 10 mg/kg/day
	Maximum dose: 600 mg
Three months of rifapentine and isoniazid taken once weekly	Rifapentine: 10-14 kg: 300 mg
	14.1 to 25 kg: 450 mg

While screening and diagnosis are crucial to LTBI management, another critical factor is to know how and when to offer the treatment to increase its acceptance by the target populace. Because it has been found from various studies that people start the treatment but fail to follow up and complete the regimen, multiple factors that seem to be contributing to it can be the unpredictability of treatment consequences in the form of fear of harmful effects, lack of knowledge of the condition, hidden costs, and social factors hindering them from keeping up with follow-ups, especially in the third world countries [20]. Most of the geographical sectors suffering from high TB index have unfortunately limited resources to combat the challenges of infectious diseases. Although treatment of the index case is done in the best possible way, contact tracing, screening, and identifying high-risk populations are not carried out [21]. Bacille Calmette-Guerin (BCG) vaccination helps reduce the severity and complications of TB disease, especially among younger people. Still, it is of no use in preventing illness or reactivation from latent infection at any time later in life. In a study done by Arguello Perez et al., it was proposed as one of the factors that may be responsible for the hindrance of completion of the treatment as BCG-vaccinated people may think of themselves as having a low chance of progressing to active disease and thus not be interested in the treatment [20].

Side effects of different LTBI regimens

In 2011, Sterling et al. conducted an open-label prospective RCT comparing three months of once weekly rifapentine + isoniazid given under DOT and nine months of daily self-administered isoniazid in age population two years and above with the aim of recording confirmed cases of TB from affected LTBI patients during follow up period and also to find completion rates in each regimen and reported adverse effects that lead to permanent discontinuation. It was found that rifapentine and isoniazid for three months and isoniazid for nine months were equally effective. The new cases of TB in the 9INH group were double compared to the 3HP group. In the 3HP group, 3.7% of patients permanently left therapy due to an adverse event which was lower than that of the INH group (4.9%) [3]; conclusions were close to Sharma et al.'s systematic review [5]. The completion rate was more remarkable in 3HP, and the toxicity profile was the same in both [3]. These findings were also analogous to Sun et al.'s RCT having the same comparator groups, with the 3HP group more likely to experience mild ADRs and resolved on their own or after treatment. The incidence of hepatotoxicity was low [1,5]. Considering the low potential of hepatotoxicity of the 3HP regimen compared to the 6-9 INH regimen, it naturally makes it a preferable option for patients with positive hepatitis C virus status who are already prone to have a high threshold for liver dysfunction [22]. Regular and multiple checking of liver function tests (LFTs) have demonstrated a low grade (grades 1 and 2) rise of bilirubin. However, the highest increase of aminotransferases was reported in the INH group (28%), then the rifampin group (24%), and most minor in the 3HP group (7%) [20], The rise in LFTs resulted in the discontinuation of the treatment multiple times, a finding similar to Sterling et al. [3].

In another retrospective observational study done by Fröberg et al. on HIV-negative migrants, the most common adverse events of the 3HP regimen found were nausea, stomach ache, headache, rash, vertigo, and flu-like illness. Only a single case of acute kidney injury was found highly likely due to rifapentine. Although it was a single case, it still needs to be taken into consideration [13,23]. An RCT in 2015 found similar results [24], reporting female sex, elderly, low BMI, and a white race to be highly likely of developing side effects [23]. While safety should be the first consideration while choosing any regimen, extreme caution and monitoring are needed when using 3HP in the elderly population (aged 50-70 years), considering their vulnerability and immunocompromised status [25].

Treating active TB during pregnancy benefits both the mother and the baby by decreasing the adverse outcomes like early fetal demise, pre-eclampsia, preterm labor, and low birth weight, but it is a debatable issue if treatment for LTBI should be given during pregnancy, as it is an immunocompromised state making them susceptible for active TB disease [26]. For the reproductive age group of women, six to nine months of isoniazid is the preferred regimen as it has been found to be not associated with adverse pregnancy outcomes even if given during the first three months of pregnancy, whereas the isoniazid rifapentine regimen remains to be a category C drug where it can only be used in pregnancy if benefits outweigh potential risks as enough reliable data on human studies is not available [26].

In general, children can tolerate LTBI regimens containing short course isoniazid and rifamycin to a much better extent [12,27,28]. Hepatotoxicity attributed to the long-duration isoniazid regimen was not reported in children in a multicentre randomized control trial and this study, children of age 2-17 years were also found to tolerate 3HP regimen quite well [27]. A systematic review using three months of daily isoniazid and rifampin in children less than 15 years of age as a trial drug [12] and an RCT using daily rifampin for four months in the 0-17 years of age population group concluded that they have a safety profile similar to 6H [28] but a better compliance and completion rate than 6INH. Moreover, rifampin is available in a kid-appropriate palatable formulation that gives it an added benefit over rifamycin [12,28].

Completion rates of different LTBI regimens

A simple dosing schedule, shorter treatment duration, lesser side effect profile, comparative safety, and decreased chances of liver damage make rifampin a better alternative to extended therapy isoniazid, which may lead to treatment discontinuation contributing to isoniazid resistance [29,30].

Completing the prescribed drug course as advised and keeping up with follow-ups for regular monitoring can be a challenge for some patients. They may tend to stop the treatment as soon as the improvement occurs and symptoms disappear. Analysis of completion rates shows that short treatment durations like three to four months promise more excellent completion rates [4,5,9,10]. An open-label RCT conducted in 2021 found similar completion rates between 3HP and daily isoniazid and rifampin [6]. Treatment completion in a program setting with 3HP was more than 4RFMP (79% vs. 70%). Though a difference was found but not very significant [9], findings analogous to a study done by Schmitt et al. in a correctional facility make it a potentially promising alternative compared to the standard 9H regimen [31]. However elderly population (greater than 80 years of age) was found to have a noticeable interruption rate even with the shorter regimens. One of the factors linked to treatment interruption was severe adverse drug reactions (ADRs) [32].

Patients' hesitation, who is not related to the health field or do not have a proper understanding of the disease, to accept and comply with long treatment durations is somewhat understandable. But a retrospective analysis of medical records shows a decreased acceptance rate for LTBI treatment even among health care workers. Among those who initiated, the completion rate was 69% [20]. This is similar to a retrospective study conducted by Han et al., where the completion rate was 68.2% [33]. Most of those who did not complete the treatment had an unspecified cause other than drug-related side effects [33]. Better understanding and knowledge of the disease and its consequences with and without the treatment and offering all available regimens for the condition to the eligible population can significantly improve the acceptance and completion rate [34].

It is common to observe that the length of the treatment and completion rates are inversely related [35]. 6-9 INH report around 50% completion rate [35], whereas three to four months of rifampin have around 85-90%. A similar high completion rate can be found with 3HP and 4RFMP regimens [35,36]. Another vital element that needs attention is DOT vs. SAD program condition. CDC approves 3HP only under the DOT program, but a clinical trial was done in the United States, Spain, and Hong Kong tested giving the drug through the SAD program testing its effect on compliance and found a rise in completion rates [37]. Hence making it a potentially acceptable strategy in the future during circumstances when DOT is not an option [37]. In contrast, Njie et al. concluded the 3HP regimen to have a high completion rate compared to other regimens with DOT rather than SAT [2].

Factors that can improve the acceptability of treatment and how adherence can be assured by keeping the patient's comfort in check are critical factors for treatment completion that should be researched more. For instance, instead of in-person DOT, observation through webcam/video (VDOT) is found to have a higher treatment completion rate. It was an intervention that was previously used for monitoring patients with active TB disease. It is time-saving, protective for TB treatment staff members, and easy to ensure compliance and improve the treatment outcome for patients with LTBI [38,39]. While VDOT has proven its benefits, other interventions like two-way SMS between the patient care provider did not have any added benefits compared to standard LTBI treatment care [40].

Efficacy of different LTBI regimens

Efficacy was measured by following the sample population given any of the treatments for latent tuberculosis and monitoring them for the activation of latent infection. Our research has found strong evidence for the efficacy and feasibility of rifamycin regimens with comparable benefits to traditional INH regimens [3,4,5,41,42]. The 2020 treatment guidelines for LTBI prefer rifamycin-based treatment regimens over 6-9 INH for suitable patients who can tolerate the drugs well and are not taking any other drugs that may cause the drug-to-drug interaction. They also recommend 6INH as a replaceable option for those who can not take rifamycin, particularly in HIV-negative persons. In 2003, CDC and ATS recommended against the use of two months of rifampin and pyrazinamide because it was linked to a high risk of hepatotoxicity [19]. The updated guidelines can help community policymakers, clinicians, and health provider organizations a great deal by keeping in mind the limitations and conditions applied, which are they apply to low TB incidence areas that are not resistant to isoniazid and rifampin [43].

Limitations

The factors that may have limited this study to an extent are probably, heterogeneity of inclusion criteria, different time frames of follow-up periods in which new cases of active tuberculosis were looked at, and different criteria to check for the completion of treatment.

## Conclusions

This study's effectiveness, safety profile, and tendency to complete the new rifamycin-based regimen and traditional isoniazid-only regimen were studied. Overall, the efficacy of new rifamycin courses is equal to isoniazid-based courses. No increased new cases of TB were noticed with the latest regimens. Hepatotoxicity is the most common side effect linked to more extended INH regimens, whereas adverse effects related to rifamycin are short-lived, quickly resolved, and lesser in severity but are one of the chief contributing factors to discontinuing the therapy. The completion rate is much better with short-duration courses compared to longer ones. 3HP has proved to be an excellent regimen for LTBI patients with comparable effectiveness, good safety profile, and above satisfactory completion rates, but it is not very commonly used worldwide and is given only under the directly observed therapy program. More clinical trials looking into its effectiveness under the "self-administered therapy" program should be part of the plan in the future, which is cost-effective for both the health units and patients and more convenient. But first, its safety under "SAT" should be thoroughly investigated. Also, more research focusing on the factors causing hindrance to compliance, apart from the long course of treatment, should be studied.
